# ﻿A new marine epipsammic diatom species, *Ambodajingensis* sp. nov. (Bacillariophyceae), from the coast of Southeast China

**DOI:** 10.3897/phytokeys.210.90876

**Published:** 2022-09-28

**Authors:** Honghan Liu, Zhen Wang, Weiwei Wu, Chenhong Li, Jiawei Zhang, Yahui Gao, Xuesong Li, Lin Sun, Junrong Liang, Jun Zhang, Changping Chen

**Affiliations:** 1 Key Laboratory of Ministry of Education for Coastal and Wetland Ecosystems and School of Life Sciences, Xiamen University, Xiamen 361102, China Xiamen University Xiamen China; 2 State Key Laboratory of Marine Environmental Science, Xiamen University, Xiamen 361102, China Xiamen University Xiamen China

**Keywords:** *Ambo*, China, Fujian Province, intertidal zone, new species

## Abstract

*Ambodajingensis* HH Liu, Z Wang, YH Gao & CP Chen, **sp. nov.** is described as a new species in samples collected from sand grains at Dajing Beach, Ningde City, Fujian Province, China. Morphological details of the new species with respect to valve shape, size and valve ultrastructure are presented based on light microscopy (LM) and scanning electron microscopy (SEM). The main features of *Ambodajingensis* under a light microscope are elongated elliptic valves with rounded apices, two internal costae on the valve and rectangular in girdle views. SEM observation showed that externally, the frustules are comprised of two valves with a relatively deep mantle and a transition between the valve faces. Small, flabelliform spines are present along the valve margin. Internally, the valves are divided into three sectors by robust costae, which penetrate the whole valve lumen and are thickest at the mantle interior and thinner toward the center. The sternum is narrow and linear, visible only in the valve apex, set off by costae. The striae are comprised of small, round areolae and they are parallel in the middle to slightly radiate at the apices. The new species is compared with other species in the genus *Ambo*.

## ﻿Introduction

The genus *Ambo* Witkowski, Ashworth, Lange-Bertalot & G. Klein is a recently established genus, with three species transferred and one species newly described by Witkowski et al. (2020). Witkowski et al. (2020) made new combinations for *Anaulusbalticus*, *Anaulussimonsenii*, and *Plagiogrammatenuissimum* and added a new species for *A.galaeciae*. The type species selected for the new genus was *Ambotenuissimum*, based on material from Lago de Maracaibo (Venezuela), the western Indian Ocean and the east coast of South Africa, and later also found to occur in the western Pacific margin. The genus *Ambo* is comprised of small taxa with internal costae across the valves. By scanning electron microscopy (**SEM**) observations, the unique features of the genus *Ambo* were revealed: hyaline valve center and areolated apices and the absence of rimoportulae.

Observations of the valve structure in *Ambo* species indicated that the costae across the valves were distinct under light microscopy (**LM**). However, in some small diatoms, with the exception of costae, a few features resolvable with LM could cause confusion in the identification and classification of these diatoms. In nonpennates and araphid diatoms, the genera *Eunotogramma* (Ashworth et al. 2015), *Anaulus* (Odebrecht et al. 2014; Franco et al. 2018) and *Plagiogramma* (Kaczmarska et al. 2017; Li et al. 2021) bear internal transverse costae. With the re-examination of some small species, i.e., *Anaulusbalticus*, *Anaulussimonsenii* and *Plagiogrammatenuissimum*, in these genera through the use of the scanning electron microscope, the valve ultrastructures in these species were revealed, including symmetric valves without rimoportula, a hyaline area in the central part of the valve and unperforated girdle bands (Witkowski et al. 2020). Therefore, the new genus *Ambo* was described by Witkowski et al. (2020) to accommodate these species. Unlike *Ambo*, the genus *Eunotogramma* has an asymmetric valve, rimoportula, variable costae and parallel striae (Ross and Sims 1972; Amspoker 2016). Moreover, both *Anaulus* and *Eunotogramma* have broad girdles comprised of numerous perforated bands (Drebes and Schulz 1989) compared to plain girdle bands in the genus *Ambo*.

Here, we describe a new species, *Ambodajingensis*, with few features resolvable with light microscopy (LM) other than internal costae across the valves. The cultured material also allowed us to examine the internal costa-bearing taxa using molecular tools, such as DNA sequence phylogenetics, to investigate the evolutionary relationship with other related species and their relationships. The molecular data, in combination with ultrastructural evidence provided by SEM, also allowed us to determine the classification of the new species.

## ﻿Materials and methods

**Figure 1.**
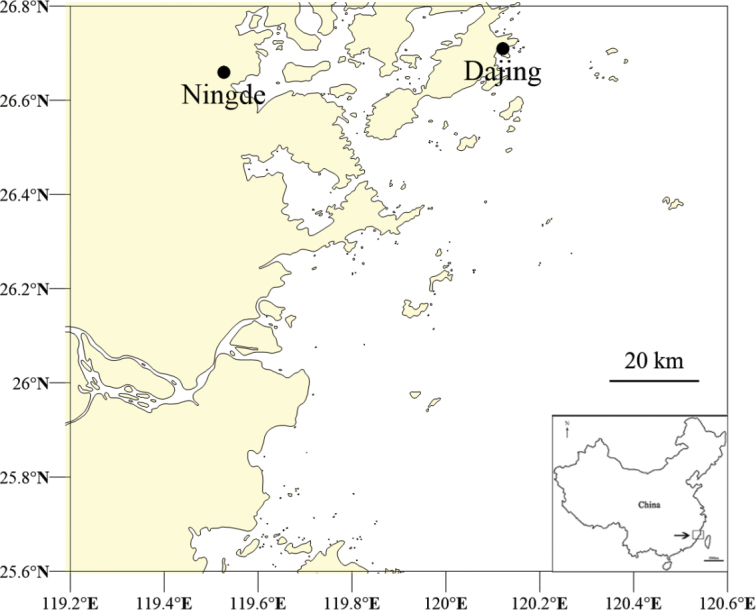
Maps with the geographic location of the study area.

The samples were collected from the intertidal zone on Dajing Beach, Ningde City, Fujian Province, China (26°42'34"N, 120°7'17"E) (Fig. 1) in May 2018. The sample site is located in a subtropical monsoon humid climate zone. The beach covers an area of approximately 0.6 km^2^. The average annual temperature is 18.6 °C, and the average annual precipitation is 1100~1800 mm.

The sand grains were collected and stored at low temperature until brought back to the laboratory. The diatoms attached to the sand particles were treated by ultrasonic waves and cultured through the pore plate dilution method, and the single high-density diatom solution was gradually expanded (Zhang et al. 2020). The diatom solution was acidified and washed 8 to 10 times with distilled water until the pH of the sample was nearly neutral (Li et al. 2022). The cleaned material was air-dried onto coverslips, and these were mounted on glass slides using Naphrax for light microscopy (Al-Handal et al. 2021; Lobban and Prelosky 2022). The diatoms were examined and identified using a Leica DM48 microscope (Leica, Germany) with a Leica Application Suite X camera (100× objective) system. The cleaned material was air-dried onto membrane filters, mounted onto aluminum stubs and coated with gold palladium for scanning electron microscopy (SEM). SEM observations were made using a JSM-6390LV scanning electron microscope at an accelerating voltage of 10 kV (Lin et al. 2022). Other portions of the diatom sample were fixed by adding glutaraldehyde at a concentration of 2.5% for 30 min at 4 °C, then rinsed with distilled water and attached to a glass coverslip. They were then dehydrated through 30%, 50%, 70%, 80%, 90%, 95% and 100% alcohol series and critical point dried (Leica CPD300) for SEM observation (Majewska et al. 2017). Valve dimensions and density of the striae were measured using LM and SEM images of 30 valves. The diatom morphological terminology follows Ross and Sims (1972), Round et al. (1990), Witkowski et al. (2020) and Van de Vijver et al. (2021). Samples are housed in the Biology Department Herbarium, Xiamen University (AU), China.

### ﻿DNA extraction and sequencing

DNA was extracted from the cultured materials. Diatom cells were pelleted in a Fresco17 refrigerated superspeed centrifuge (Thermo Fisher Scientific, USA) for 10 min at 7649× g from a culture in the late logarithmic phase of growth. The product instructions of the Steady Pure Plant Genomic DNA Kit were followed to extract genomic DNA from monoclonal diatom cells that had been cultured for two weeks. Gel electrophoresis (1% agarose gel) and microspectrophotometry were used to determine the purity and concentration of the extracted DNA. The DNA samples were stored at –20 °C before the polymerase chain reaction (PCR). PCR using eukaryotic primers R1F 5’-TTAAGGAGAAATAAATGTCTCAATCTG-3’ and R1R 5’-GCGAAATCAGCT GTATCTGTWG- 3’ was performed on the total DNA extracts. Refer to the primers of Alverson et al. (2007) and Ruck and Theriot et al. (2011) to amplify the *rbc*L gene fragment.

Polymerase chain reaction was performed with the premixed mix PrimeSTAR to amplify the *rbc*L gene fragment. The amplification conditions of *rbc*L were: initial denaturation at 95 °C for 5 min, denaturation at 95 °C for 30 s, annealing at 55 °C for 30 s, and extension at 72 °C for 60 s, for a total of 36 cycles, and a final extension at 12 °C for 15 min. The PCR products were detected by 1% agarose gel, purified using a SanPrep column PCR product purification kit, and sent to Sangon Sequencing Company for sequencing.

### ﻿Phylogenetic analysis

Phylogenetic analysis of the molecular data was conducted using the *rbc*L dataset. For DNA barcoding of diatoms, Hamsher et al. (2011) showed that the *rbc*L gene could be well selected based on its variance (Samanta and Bhadury 2018). Through BLAST in the GenBank database to compare the *rbc*L gene sequence obtained from this experiment, we downloaded 56 strains belonging to the araphid group in this study and the strain of *Bolidomonaspacifica* was chosen as the outgroup taxon. Then, the DNA sequence obtained from this experiment were merged with the sequence of the outgroup taxon. The resulting sequences were checked and first aligned using the mafft V7.110 online program (http://mafft.cbrc.jp/alignment/server/) and the default settings. We manually checked the alignment using BioEdit v.7.0.9 (Hall 1999). The maximum likelihood (ML) analysis was carried out by Raxml V7.2.6 (Stamatakis and Alachiotis 2010) using the Model GTRMM in T-rex web servers (Alix et al. 2012). The bootstrap values were obtained by making 1000 replicates of the ML analyses for each branch node of the phylogenetic tree (Guindon et al. 2010).

## ﻿Results

### ﻿ Ambo dajingensis

Taxon classificationPlantaeRhabdonematalesGrammatophoraceae

HH Liu, Z Wang, YH Gao & CP Chen sp. nov.

61E9F804-E0AC-5924-8751-A40915BCB6A9

Figs 2 , 3 , 4

#### Holotype

**Figure 2.**
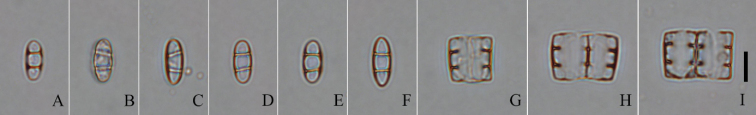
Light micrographs (LM) of *Ambodajingensis* sp. nov. **A–F** valve showing the size and shape **G–I** girdle view. Scale bar: 5 µm.

**(here designated)**: slide DJ1805 (AU, Biology Department Herbarium, Xiamen University). Fig. 2E represents holotype.

#### Type locality.

Sand beach in Dajing County, Fujian Province, China, 26°42'34"N, 120°7'17"E, *leg.* Zhen Wang in May 2018

#### Etymology.

The epithet “*dajingensis*” refers to the site where this specimen was collected.

#### Description.

**LM observations** (Fig. 2A–I): The cells are connected to each other to form short chains and they live in groups. The girdle is comprised of several plain and open bands. The valves are linear elliptic with two costae that are straight or slightly curved to the outside and protrude deeply into the valve. The valve is 6–10 μm long and 2.4–3 μm wide.

**Figure 3.**
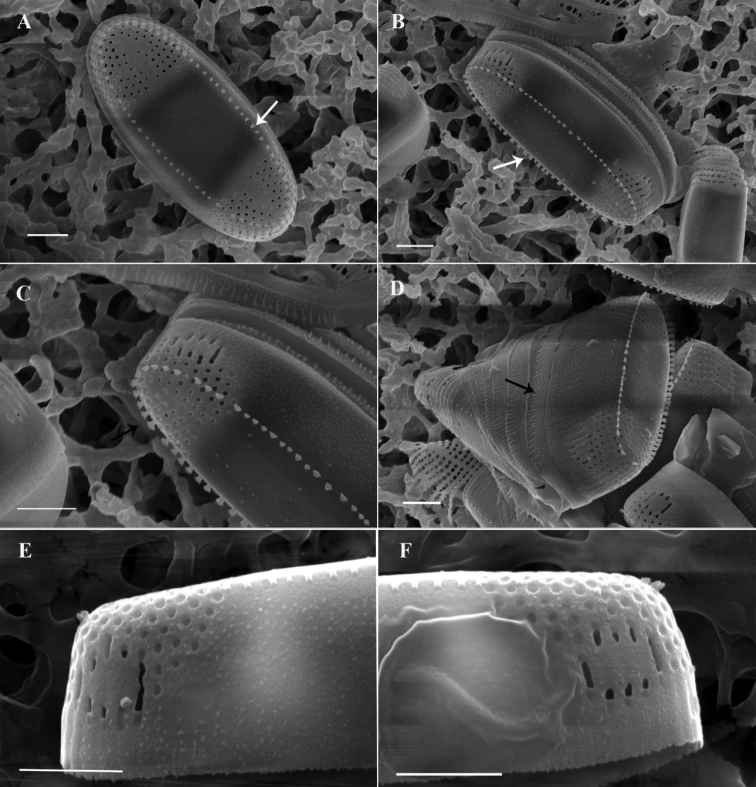
Scanning electron microscopy (SEM) images of *Ambodajingensis* sp. nov. in an external view of the entire valve **A, B** external valve view of the whole specimen. There is a hyaline valve face over most of the length and transapical striae develop only at the valve apex, with a weakly expressed sternum at the apices **C, E, F** fan-shaped spines (arrows) are arranged along the valve margin, there is striation, a strongly expressed sternum on the valve apical part, a small circular striated area structure on the mantle apex **D** the girdle bands. Scale bar: 1 µm.

**Figure 4.**
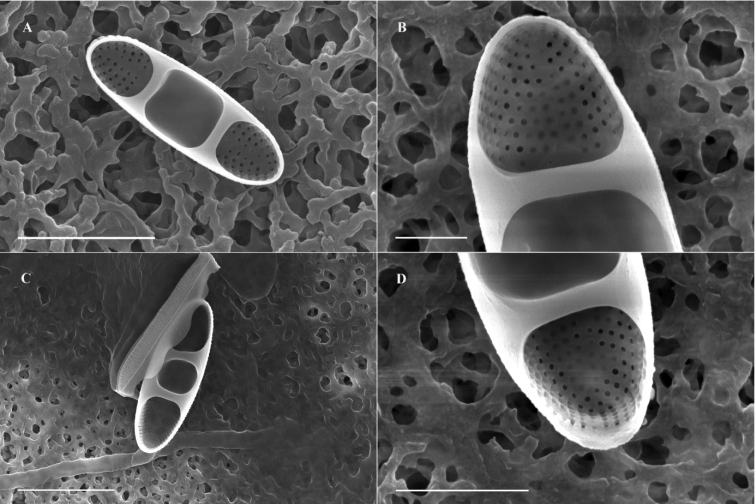
Scanning electron microscopy (SEM) images of *Ambodajingensis* sp. nov. in an internal view of the entire valve **A, C** internal valve view of the whole specimen **B, D** the presence of costae (arrows) and the areolation. Scale bars: 3 µm (**A**); 1 µm (**B**); 5 µm (**C**); 2 µm (**D**).

**SEM observations** (Figs 3, 4): Through the observation of the samples treated by the critical point drying method, the cells on the girdle band valve are rectangular (Fig. 3A), with a wrapped layer of membrane. Several girdle bands (Fig. 3D) are connected between the epitheca and hypotheca valves. The girdle band has fine vertical lines (Fig. 3D) but no obvious spot pattern. Areolae can be observed at both ends of the valve, and the area of the areolae extends to the sternum, but there are no areolae or other cell structures in the middle of the cell (Fig. 3A, B). There is a circle of spines for cell connection at the edge of the valve (Fig. 3A–D). The single row of slightly radial striae is distributed at both ends of the cell (Fig. 3A). Observation of the samples processed by the acidification method showed striae at both ends of the cells and a narrow sternum in the center of the cells in the area of striae, but the sterna were not obvious. To the two ends of the valve, the striae at both ends of the valve extended from the valve in a single row, and the number of striae was 50–60 in 10 μm. The areolae on the valve are round, small, simple, and have no complex structure, such as a cribrum. A small circular area free of areolae is distinctly set off from the striae, and can be observed at the apical part of mantle area with several elongate areolae following the general pattern of the mantle (Fig. 3E, F). On the edge of the valve, there is a row of densely distributed small spines.

Internally, the valves are divided into three sectors by robust costae (Fig. 4A, C). The interior of the valve face is flat, the transition from the valve face to the mantle is abrupt (Fig. 4A). The transapical striae are composed internally of small, round areolae, and they are parallel in the middle to slightly radiate at the apices. The sternum is narrow and linear, set off by costae (Fig. 4A).

#### Distribution.

Marine, coastal. Collected from Dajing beach on sandy shores. Changchun Town, Xiapu, Ningde City, Fujian Province.

##### ﻿Molecular phylogeny

**Figure 5.**
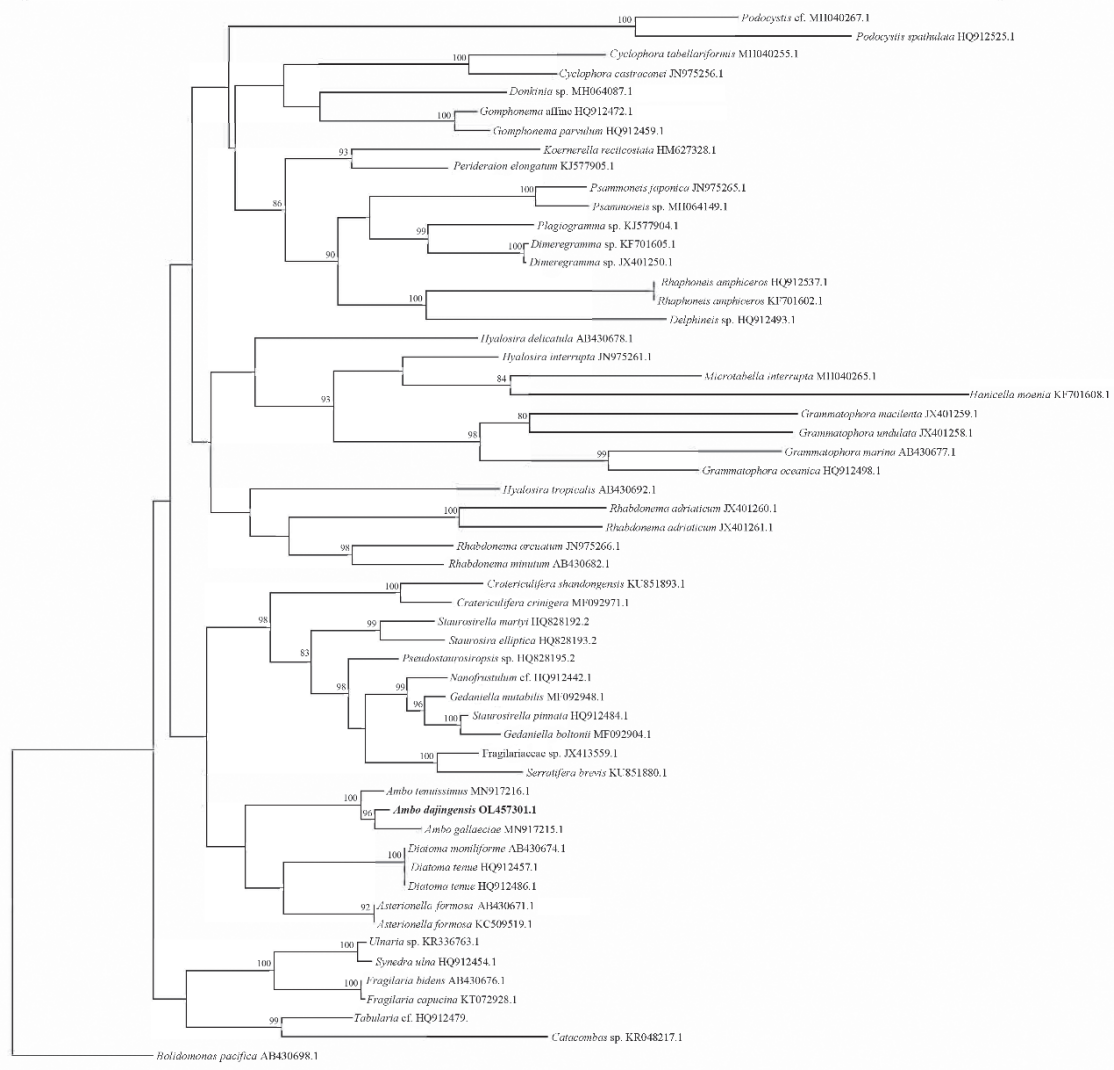
Maximum-Likelihood Phylogenies tree. Maximum-Likelihood Phylogenies (MLP) tree (based on analysis of the *rbc*L dataset) of mediophycean strains related to *Ambo*.

Phylogenetic analysis of molecular data was conducted using a *rbc*L-gene dataset. The tree inferred from Maximum-Likelihood Phylogenies (MLP) analysis (Fig. 5) of the concatenated *rbc*L-gene dataset recovered the genus *Ambo* (represented by *A.tenuissimus*, *Ambodajingensis* and *A.gallaeciae*) within the araphid diatoms, sister (bootstrap support [bs] = 100%) to a clade with *Diatomamoniliforma*, *Diatomatenue*, and *Asterionellaformosa*. *Ambodajingensis* and *A.gallaeciae* were sister to a clade, but with low support. The phylogenetic relationships of the new species were sustained by genetic distance estimation. The sequences generated in the study were deposited in GenBank under access number OL457301.1.

## ﻿Discussion

The characteristics of the genus *Ambo* were also observed in our samples. These features include 1) girdles consisting of several plain and open bands, 3) each costa near the apices, 4) areolae restricted to the ends of the valve face and absent at the central area, and 5) rimoportula absent.

Phylogenetic analysis of the *rbc*L sequences strongly supports our suggestion, based on morphological characteristics, that *A.dajingensis* is a new species for this genus. *A.dajingensis* and *A.gallaeciae* were positioned in a clade, which was sister to *A.tenuissimus* with a high support (bootstrap support = 100%). Our results revealed that the genus *Ambo* was sister to a clade with *Asterionella* and *Diatoma*. However, a much lower level of support (bootstrap support < 50) suggested that putting *Ambo* in Tabellariaceae could be difficult. Combining three-gene (nuclearencoded small subunit (SSU) rRNA, *rbc*L and plastid-encoded psbC) analysis, Witkowski et al. (2020) suggested that *Ambo* was sister to the Grammatophoraceae (*Hyalosira*, *Microtabella*, *Hanicella*, and *Grammatophora*) rather than Tabellariaceae (*Asterionella* and *Diatoma*). Lobban et al. (2021) also concluded that *Ambo* was sister to the Grammatophoraceae with high support (bootstrap support = 100%, posterior probability= 1.0).

A comparison of *Ambodajingensis* with other morphologically similar species is shown in Table 1. The length and width of *A.dajingensis* are similar to those of *A.balticus*. The valves of *Ambodajingensis* are 6–10 μm long and 2.4–3 μm wide, and *Ambobalticus* valves are 5.5–14 μm long and 2.5–4 μm wide. However, *A.dajingensis* differs from *A.balticus* in several aspects: the general valve outline, which is more elliptical and narrower; the valve, which is symmetrical about the apical axis; and the number of striae, which is higher. Moreover, the presence of fan-shaped spines arranged along the valve margin and growing independently is another important feature for *A.dajingensis* (Fig. 3A–D), comparing with small and globular spines present along the valve margin, sometimes growing together into a ridge-like structure in *A.balticus* (Witkowski et al. 2020). Sternum in *A.dajingensis* is distinct and somewhat far from the apices (Fig. 3A), while Sternum in *A.balticus* is indistinct, and well expressed at apices. *A.dajingensis* shows some similarities to *A.tenuissimus*, which has comparable striation, a linear and narrow sternum, and an abrupt mantle with a small circular area free of areolae distinctly set off from the striae and surrounded by several elongate areolae.

**Table 1.** Morphometric characteristics of *Ambodajingensis* and comparison with other *Ambo* taxa (Witkowski et al. 2020).

Species	Valve outline	Valve apices	Spines	Valve length (μm)	Valve width (μm)	Striae (10 μm)	Areolae (10 μm)
*Ambodajingensis*	elongated and elliptic	rounded	fan-shaped	6–10	2.4–3	50–60	50–60
*Ambobalticus*	linear to linear lanceolate	narrow and expressed	small, globular	5.5–14	2.5–4	50	60
*Ambogallaeciae*	linear to linear elliptic	broadly rounded	small, irregularly distributed globular	6.5–8	3.5–4.5	55	70–80
*Ambosimonsenii*	narrowly linear-lanceolate with protracted	rounded	No data	4.5–15.5	1–2.1	80–100	120
*Ambotenuissimus*	linear elliptic	rounded	small, globular	9–15	2	48–60	70–80

However, they differ in many other features. The first is the valve outline, which is linear elliptic in *A.tenuissimus* but elliptic lanceolate in our new species. Moreover, in *A.dajingensis*, the spines are fan-shaped along the valve margin, not growing together into a continuous ridge (Fig. 3A–D), while in *A.tenuissimus*, the spines are small and globular along the valve margin, sometimes growing together into a continuous ridge (Witkowski et al. 2020). *A.dajingensis* is different from *A.tenuissimus* in areolae density (50–60 in 10 μm vs. 70–80 in 10 μm) (Table 1).

*A.dajingensis* is distinguished from *A.gallaeciae* by LM using size dimensions. *A.dajingensis* is 6–10 μm long and 2.4–3 μm wide with rounded apices, whereas *A.gallaeciae* is 6.5–8.0 μm long and 3.5–4.5 μm wide with broadly rounded apices. In SEM, a relatively deep mantle and an abrupt transition between the valve face and the mantle of *A.dajingensis* serve to distinguish this species from a relatively shallow mantle and a gradual transition between the valve face and the mantle of *A.gallaeciae*. Moreover, in *A.gallaeciae*, the spines are small, globular and irregularly distributed at the transition from the valve margin to the mantle, whereas in *A.dajingensis*, they are fan-shaped along the valve margin. The differences between *A.dajingensis* and *A.simonsenii* are based on the valve width (2.4–3 μm vs. 1–2.1 μm), stria density (50–60 in 10 μm vs. 80–100 in 10 μm) and areolae density (50–60 in 10 μm vs. 120 in 10 μm). Additionally, the presence *vs.* absence of spines on the valve surface also distinguish these species.

All the diatoms of *Ambo* genus had been found in the marine environment, and they are widely distributed in different regions of the world. *Ambobalticus* were observed in the Western Baltic Sea, Africa East coast, Sodwana Bay, Pacific Ocean, etc. *Ambosimonsenii* were in the western Baltic Sea, Disko Bay, North Sea, etc. *Ambotenuissimus* was reported from Venezuela, the Indian Ocean, the Yellow Sea coast, China, etc. *Ambogallaeciae* are known from the Atlantic coast of NW Spain. The samples of this genus were collected from Dajing Beach, Ningde City, Fujian Province. *Ambodajingensis* sp. nov. is a marine epipsammic araphid diatom.

The newly documented species in the present study were collected from sand, suggesting that the diversity of tiny araphid taxa is understudied in these habitats and remains to be further explored. The diversity of small-celled diatoms is easily ignored when observed under LM, in which distinguishing characteristics are difficult to resolve. On the basis of our study, we suggest that among the small-celled taxa, the diversity in their ultrastructural morphology and genetic data reflect a great deal of taxonomic diversity, despite their small valves, overlapped size dimension and striae density. It is entirely possible that this taxonomic diversity also reflects a strong diversification across ecological habitats. When encountering small diatoms, it is necessary to focus on their ultramorphological (examined with SEM) or phylogenetic differences, which are likely diverged by some specific type of environment and should not be ignored.

## Supplementary Material

XML Treatment for Ambo dajingensis
